# Infrared Thermal Imaging as a Predictor of Lumbar Paravertebral Block Effectiveness in Cattle

**DOI:** 10.3390/ani16010127

**Published:** 2026-01-02

**Authors:** Jaime Viscasillas, Elsa Rave, Ariel Cañón-Pérez, María De Los Reyes Marti-Scharfhausen, Eva Zoe Hernández-Magaña, Agustín Martínez, José Ignacio Redondo, Angel García-Muñoz

**Affiliations:** 1 AniCura Valencia Sur Hospital, Av. Picassent 28, Silla, 46460 Valencia, Spain; 2 UNIVET, 347 Rue des Corvées, 38660 Le Touvet, France; elsarave.vet@gmail.com; 3 Experimental Surgery Unit, Vall D’Hebron Institut de Recerca (VHIR), Pg. de la Vall D’Hebron 129, Horta-Guinardó, 08035 Barcelona, Spain; ariel.canon@vhir.org; 4 AniCura Indautxu Hospital, San Mamés Zumarkalea 36–38, 48010 Bilbao, Spain; reyesvet92@gmail.com; 5 Departamento Medicina y Cirugía Animal, Facultad de Veterinaria, Universidad Cardenal Herrera-CEU, CEU Universities, 46115 Valencia, Spain; eva.hernandezmagana@uchceu.es (E.Z.H.-M.); nacho@uchceu.es (J.I.R.); 6 AniCura Aitana Hospital, C/Xirivella 16, 46920 Valencia, Spain; agustin.martinez.vet@gmail.com; 7 Departamento Producción y Sanidad Animal, Salud Pública Veterinaria y Ciencia y Tecnología de los Alimentos (PASAPTA), Facultad de Veterinaria, Universidad Cardenal Herrera-CEU, CEU Universities, 46115 Valencia, Spain; angel@uchceu.es

**Keywords:** cattle, lumbar paravertebral block, regional anaesthesia, thermography, ultrasound

## Abstract

In the daily clinical practice of cattle, the use of locoregional anaesthesia is needed to provide analgesia during standing surgical procedures. It is important to ensure the success of the blockade before starting the surgery. One of the most used techniques is the paravertebral lumbar block. In this pilot study we evaluated the efficacy of thermography in assessing this block. For this matter, 12 cows from our university research and teaching farm, with similar characteristics, were included and in which an ultrasound-guided technique of lumbar paravertebral block (T_13_/L_1_) or (L_1_/L_2_) with lidocaine was performed. Thermal photographs were taken with a FLIR^®^ One camera at 0, 15, 30 and 45 min and at the same time a test to evaluate the response to a painful stimulus was performed in each dermatome (T_13_, L_1_, L_2_ and L_3_). The data was collected in predesigned cards and placed in the Excel programme for further statistical analysis with the R programme. The analysis determined a correlation between the increase in skin temperature of the dermatomes that had been blocked and the increase in skin temperature and the negative response to the painful stimulus test. Although the pilot study has some limitations, this allows us to assess the use of thermography as an efficient method for assessing the success of lumbar paravertebral blockade in cattle.

## Simple Summary

Locoregional anaesthesia is commonly used in cattle to provide pain relief during standing surgeries. For these procedures, it is essential to confirm that the block is effective before beginning the surgery. The lumbar paravertebral block is one of the most frequently applied techniques in cattle. In this pilot study we evaluated the efficacy of thermography in assessing this block. Twelve cows underwent an ultrasound-guided lumbar paravertebral block with lidocaine, and thermal images were taken at different time points alongside pain response tests. The results showed that an increase in skin temperature correlated with a reduced response to painful stimulation, supporting the use of thermography as a simple and efficient tool to evaluate block effectiveness in cattle.

## 1. Introduction

Locoregional anaesthesia techniques are an effective method of providing analgesia in animals. These techniques involve deposition of a local anaesthetic in close proximity to a peripheral nerve; upon contact with the nerve membrane, the drug blocks voltage-gated sodium channels, thereby inhibiting sodium influx into axons, preventing depolarisation and interrupting neural transmission [1]. This mechanism can produce complete analgesia [1]. The duration of the nerve block depends, among other factors, on the specific local anaesthetic used (e.g., lidocaine, bupivacaine, ropivacaine) and on its pharmacokinetic/physicochemical properties (e.g., lipid solubility, protein binding, etc.) [2].

Locoregional techniques are widely used in both human and veterinary anaesthesia to deliver high-quality analgesia [3,4]. Nerve blocks are frequently performed alongside general anaesthesia or sedation to facilitate procedures. When combined with general anaesthesia, they may reduce requirements for intravenous or inhalational agents, thereby lowering the risk of cardiorespiratory depression and improving recovery [5]. Likewise, when combined with sedation, they can enable standing surgery in large animals, avoiding adverse effects associated with general anaesthesia without compromising analgesia [1]. These techniques are increasingly adopted in veterinary medicine, in both small and large animals, with significant reported benefits [4].

In cattle, achieving adequate analgesia can be challenging owing to legal restrictions on drug use in food-producing species. Because cattle and their products are intended for human consumption, systemic analgesics are tightly regulated, and fewer drugs are authorised than in small animal practice. Beyond these constraints, many bovine surgeries are urgent, field procedures performed with limited infrastructure (e.g., anaesthetic machine, induction stall, reliable intubation equipment) and personnel, with restricted budgets. Locoregional anaesthesia therefore represents an excellent alternative to provide effective analgesia while minimising systemic drug administration [6].

The lumbar paravertebral block (LPVB) is widely used in cattle to provide unilateral analgesia of the flank for procedures such as caesarean section and correction of abomasal displacement; it targets the ventral rami of the T_13_–L_3_ spinal nerves and can provide both somatic (abdominal wall) and visceral (intra-abdominal organ) analgesia [7,8]. The average incidence of abomasal displacement (AD) in dairy cattle has been estimated at 1.7%, with reported values ranging from 0.3% to 6.3% [9]. According to the USDA National Animal Health Monitoring System [10], the incidence of abomasal displacement is approximately 2.2%, whereas caesarean sections account for less than 0.1%. Left abomasal displacement (LDA) therefore remains one of the most common surgical disorders in dairy cows and is typically corrected by right paralumbar laparotomy and omentopexy performed with the animal standing [11]. Although the frequency of caesarean sections has declined in recent years, they continue to represent a relevant cause of surgical intervention, with a surgical approach comparable to that employed for LDA.

Several approaches to this block have been described (proximal/Farquharson and distal/Magda/Cornell), traditionally performed ‘blind,’ relying on external landmarks. Although generally safe, severe adverse effects such as central nervous system toxicity, seizures and even death have been reported in overdose scenarios [12]. Moreover, if the injectate is deposited too far from the target nerves, the block may be incomplete or fail, compromising animal welfare and operator safety. Accordingly, accurate needle placement, depositing the drug as close as possible to the nerve while using the lowest effective volume, is crucial. Ultrasound guidance has been described for this block to improve localisation of the injection site [13] and may reduce dose-related adverse effects while enhancing efficacy [14]. The closer the injectate is to the nerve, the lower the dose typically required to produce anaesthesia and, hence, the fewer potential side effects.

Another critical consideration, especially for standing surgery under sedation, is to confirm, before incision, that the block is effective. Functionally, the lumbar paravertebral block comprises four separate injections (T_13_, L_1_, L_2_, L_3_); thus, it is essential to ensure that all targeted dermatomes are desensitised prior to surgery [4]. In current practice, assessment methods are often subjective. Common bedside indicators include development of lordosis (reflecting muscle relaxation) and palpable skin warming due to local-anaesthetic-induced vasodilation [15]. Infrared thermography (IRT) detects changes in skin surface temperature that reflect underlying alterations in blood flow and sympathetic tone, and has been proposed as a non-invasive, objective indicator of successful regional blockade [16]. In human anaesthesia, thermography has been used to detect post-block temperature changes and to help predict block success [17,18]. Moreover, most veterinary reports to date have focused on lameness or inflammation [19] rather than on using IRT as an immediate predictor of regional block efficacy; the evidence base for block-validation using thermography in large animals is therefore limited. This gap motivates evaluation of IRT specifically for LPVB assessment in cattle [20].

A practical consideration is device selection. High-end radiometric cameras (research-grade instruments) offer superior spatial resolution, thermal sensitivity and radiometric accuracy, but are costly and less practical in field settings. Recent comparative work shows that smartphone-based modules (e.g., FLIR One^®^) can detect gross thermal changes after peripheral nerve block [21], but they frequently differ in absolute temperatures from research cameras and may exhibit greater variability and drift. Thus, while smartphone IRT is promising for point-of-care screening, its quantitative reliability and the appropriate thresholds for predicting block success require validation for each species and clinical application [22].

Against this background, we conducted a pilot study to evaluate whether smartphone-based infrared thermography can anticipate the analgesic effect of an ultrasound-guided lumbar paravertebral block in cattle. Our primary hypothesis was that successful blockade would produce a measurable, dermatome-specific increase in skin temperature detectable with a smartphone thermal camera, and that this temperature rise would correlate with reduced nociceptive responses to standardised cutaneous stimulation. Establishing such a correlation could provide clinicians with a rapid, non-invasive method to confirm block success prior to surgery, and inform the design of larger validation studies.

## 2. Materials and Methods

A prospective, experimental study was conducted at the teaching and research farm of University CEU–Cardenal Herrera (Valencia, Spain). The protocol was reviewed and approved by the institutional Animal Ethics Committee (approval number 2021/VSC/PEA/0127). The ARRIVE 2.0 guidelines [23] were followed to ensure that all relevant information was included and that all stages of the study were conducted appropriately. Twelve healthy adult Holstein cows were enrolled. Animals were acclimated to handling and the experimental environment for one week before the procedure. Body condition score (BCS) was evaluated using a 5-point scale, where 1 indicates very thin and 5 indicates obese. Cows exhibiting signs of agitation, lameness, or systemic illness were excluded.

**Figure 1:**
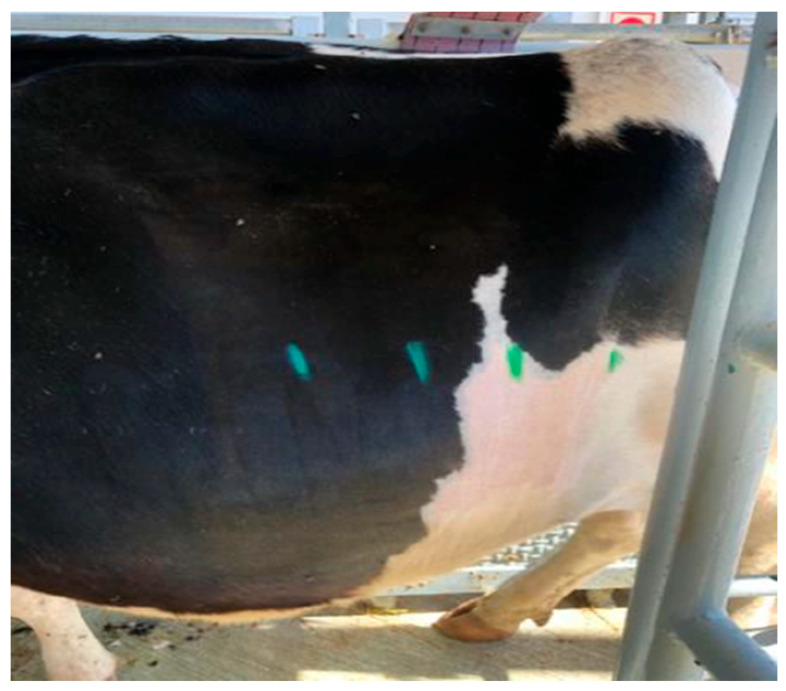
Lateral abdominal region of a study cow after hair clipping, with dermatome boundaries delineated in green.

All procedures were performed with the animals standing in individual stocks to minimise stress and movement. Hair was clipped from the dorsal lumbar region, where injections were performed, extending ventrally approximately 30 cm from the linea alba to avoid interference with ultrasound and temperature measurement. The dorsal area was disinfected with 1% chlorhexidine (Desinclor 1%, Ajalvir, Spain). A square area on the contralateral thorax was clipped as a control site. Blocks were performed on either the left or right flank depending on crush configuration. The dermatomal boundaries innervated by T_13_, L_1_, L_2_ and L_3_ [13] were outlined with a skin marker (Figure 1).

Baseline measurements comprised thermographic images of the abdominal region and of the contralateral thoracic control square. Images were acquired using a FLIR^®^ one thermal camera (Flir, Täby, Sweden) attached to an iPhone 12 mini (Apple, Zhengzhou, China) and stored on the smartphone. Device specifications were as follows: thermal resolution 160 × 120, temperature range −20 to 120 °C (up to 400 °C on Pro models), and stated accuracy ±3 °C or ±5% within the recommended operating range. Cutaneous sensation was evaluated by gentle skin pinch with a mosquito haemostat and observation of a skin-twitch response. Measurements were obtained within each dermatome at the midpoint between the dorsal midline and the linea alba, at a constant camera-to-skin distance and angle. All procedures were performed on different days within the same week and at approximately the same time of day to ensure comparable meteorological conditions.

**Figure 2:**
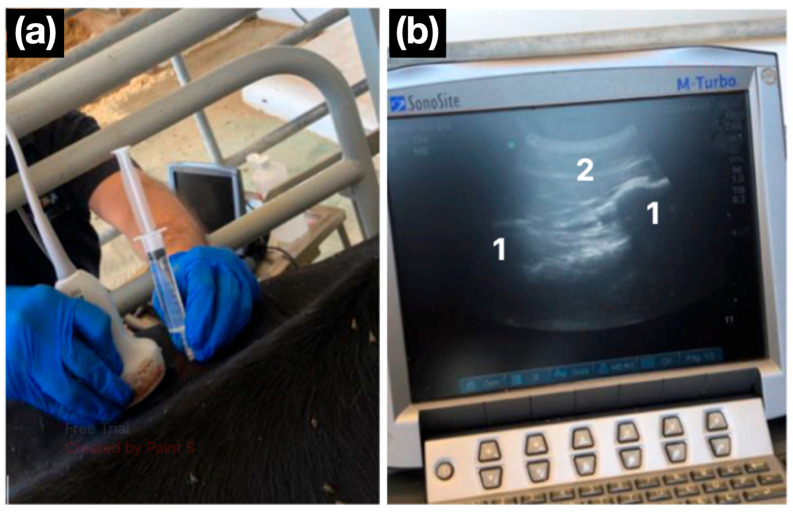
Ultrasound-guided lumbar paravertebral block. (**a**) A convex transducer is positioned in a parasagittal orientation, and the needle is advanced in a cranio-caudal direction using an in-plane approach. (**b**) The ultrasonographic image shows two flattened hyperechoic structures with posterior acoustic shadowing, corresponding to the transverse processes of the lumbar vertebrae (1). The needle is observed advancing caudally to the transverse process (2).

All the blocks were performed under ultrasound guidance using a portable unit (M-Turbo, SonoSite, Bothell, USA) with a curvilinear transducer (3–5 MHz), following the published description [13]. Bony landmarks were identified ultrasonographically: the last rib and first transverse process for T_13_, and the transverse processes of L_1_ and L_2_ for the remaining nerves. After subcutaneous infiltration of 2 mL 2% lidocaine (Lidor, VetViva Richter GmbH, Wels, Austria) to desensitise the skin and needle track, an 18 G, 90 mm Quincke spinal needle (BD-Becton and Dickinson, San Agustín de Guadalix, Spain) was advanced to the target site. Following negative aspiration, 10 mL of 2% lidocaine was injected at each target (Figure 2).

**Figure 3:**
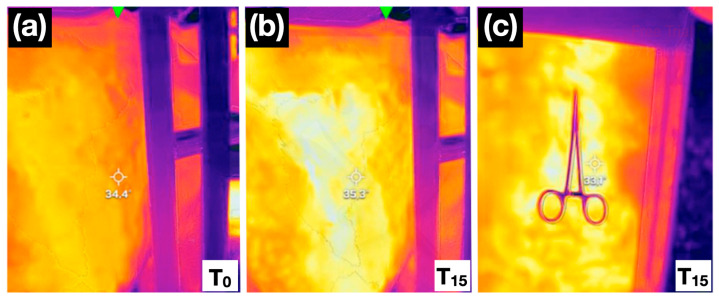
Thermal images at baseline (0 min; (**a**) and 15 min (**b**) after T_13_ and L_1_ nerve blocks, showing an increase in skin temperature. Observe the colour shift associated with increased temperature: in the first image (**a**), the evaluated area appears orange, whereas 15 min after the locoregional block (**b**), the same region displays a more yellow hue. The right-hand panel at 15 min (**c**) also shows a negative mosquito haemostat skin pinch test, with no pain-related response observed in the cow.

Each animal received two randomly selected blocks, either T_13_ and L_1_ or L_1_ and L_2_, according to a randomisation table generated in Microsoft Excel^®^ (Microsoft Corp., Redmond, WA, USA). All blocks were performed by the same experienced operator to reduce inter-individual variability (JV). Thermographic images of the abdomen and the thoracic control area were obtained at 15, 30 and 45 min after completing the blocks by another operator. All thermographic images were taken from a constant distance (1 m) and always by the same operator (ER). Finally, at the same time points, the skin-twitch response to a pinch with mosquito haemostats was reassessed (Figure 3). The pinch test was performed in every case by a third operator (AG) blinded to the identity of the blocked nerves and the thermographic images. An absence or reduction in the cutaneous twitch in the target dermatome, and/or a diminished behavioural response to a mosquito haemostat skin pinch, compared with baseline or the control area (if uncertain), was considered evidence of an effective nerve block. After data collection, cows were returned to their housing and monitored for potential adverse effects.

Following completion of all measurements, the datasets obtained by the three operators were compiled into a single Excel spreadsheet which included the following: animal identification, drug used, date, ambient temperature, side of the block, time of each injection, which nerve(s) were blocked, and the response to a nociceptive stimulus (skin pinched with a mosquito haemostats), graded as absent/mild or normal and skin temperature measured by thermography.

Statistical analyses were performed in R (version 4.2.0) (Vienna, Austria). The collected data were assessed for normality using the Shapiro–Wilk test. Body condition score was expressed as mean ± standard deviation. Temperature data were expressed as median (range) and analysed using the Friedman test. Values of *p* < 0.05 were considered statistically significant.

## 3. Results

All twelve Holstein cows tolerated handling and restraint without complications. All of them weighed approximately 600 kg with a body condition score of 2.5 ± 0.5 (1–5 scale). No sedation was required, and none of the animals exhibited overt signs of stress during the procedures. The paravertebral block was performed on the right flank in four cows and on the left flank in eight cows. The nerves targeted were T_13_ and L_1_ in seven cows, L_1_ and L_2_ in four cows, and L_1_ in one cow. No ataxia, systemic toxicity, localised swelling or other adverse effects were observed. Table 1 summarises thermography measurements and nociceptive responses over time in both blocked and non-blocked dermatomes.

**Figure 4:**
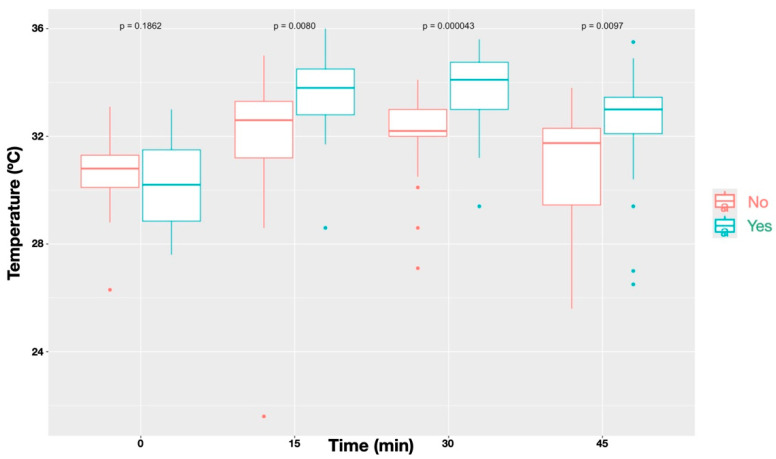
Temperature profiles over time for sites that received a block (‘Yes’) compared with those without a block (‘No’). Data represent all nerves combined. Blocked sites showed significantly different temperature patterns relative to unblocked sites (*p* < 0.001), with differences reaching statistical significance at all time points (*p* = 0.0080 at T_15_, *p* = 0.00043 at T_30_, and *p* = 0.0097 at T_45_), except before administration of the local anaesthetic block (*p* = 0.1862).

**Figure 5:**
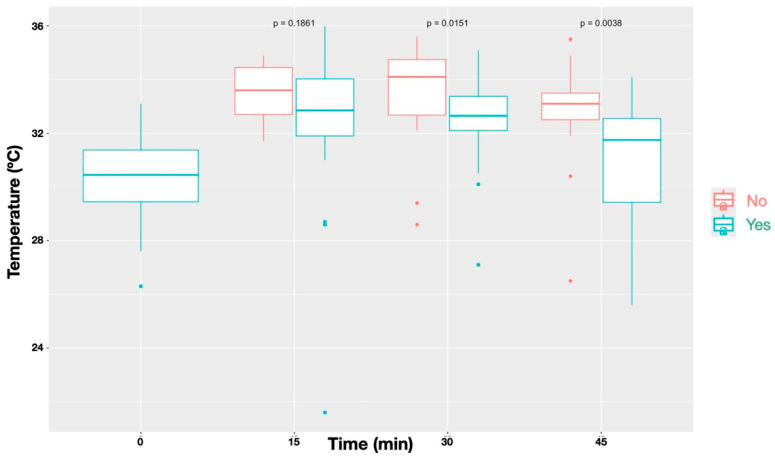
Temporal changes in skin temperature stratified by the outcome of the painful stimulus test (‘Yes’ = normal response; ‘No’ = diminished/absent response). Results represent all nerves combined. Sites with a diminished or absent response exhibited significantly higher temperatures than those with a normal response (*p* < 0.001), with differences reaching statistical significance at T_30_ (0.0151) and T_45_ (*p* = 0.0038), but not at T_15_ (*p* = 0.1862).

A statistically significant difference (*p* < 0.0001) was observed in the temperature increase between blocked and non-blocked nerves (Figure 4). Similarly, The Friedman test also revealed significantly higher temperatures in dermatomes showing an absent/diminished nociceptive response compared with those with a normal response (*p* < 0.0001; Figure 5).

**Figure 6:**
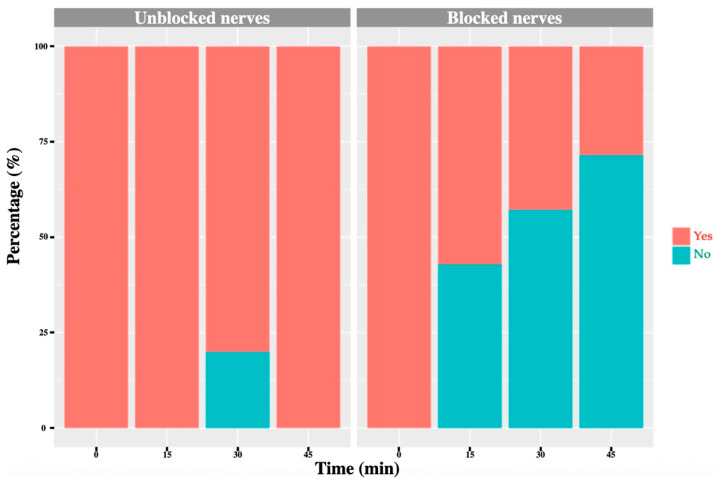
T_13_ nerve: graph showing the percentage of response to a noxious stimulus according to whether the nerve was blocked or not, and across different time points. ‘Yes’ means the animal showed a painful response. ‘No’ means the animal did not show painful response.

**Figure 7:**
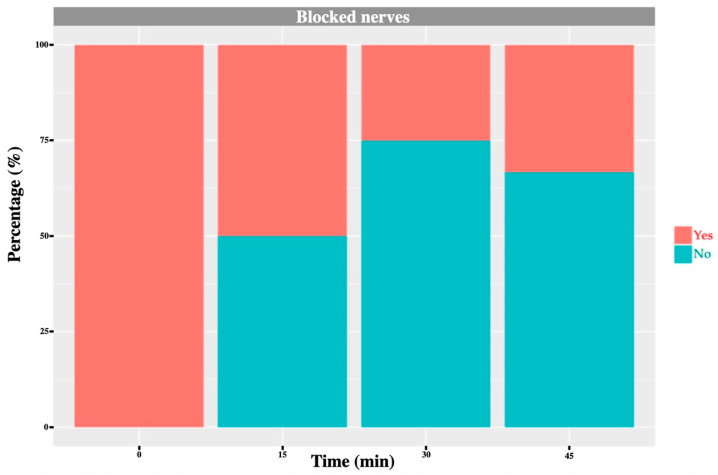
L_1_ nerve: graph showing the percentage of response to a noxious stimulus according to whether the nerve was blocked or not, and across different time points. ‘Yes’ means the animal showed a painful response. ‘No’ means the animal did not show painful response.

**Figure 8:**
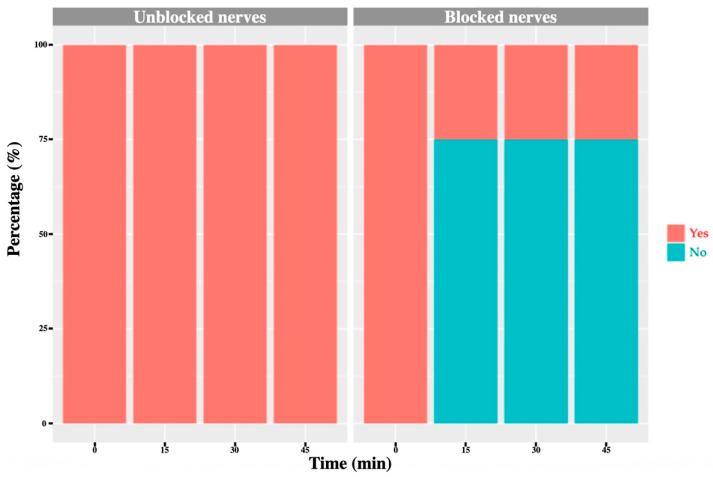
L_2_ nerve: graph showing the percentage of response to a noxious stimulus according to whether the nerve was blocked or not, and across different time points. ‘Yes’ means the animal showed a painful response. ‘No’ means the animal did not show painful response.

**Figure 9:**
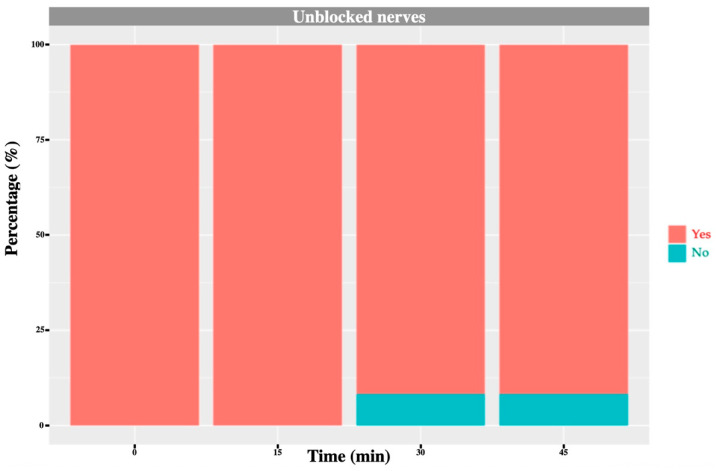
L_3_ nerve: graph showing the percentage of response to a noxious stimulus according to whether the nerve was blocked or not, and across different time points. ‘Yes’ means the animal showed a painful response. ‘No’ means the animal did not show painful response. In this case, no L_3_ nerve block was performed; however, one cow exhibited an absent/diminished response at 30 and 45 min.

Before block placement, all cows exhibited a normal, consistent skin-twitch response to mosquito haemostat pinching in each dermatome. After block administration, a reduced or absent response was observed in the majority of blocked dermatomes: 71% for T_13_, 91% for L_1_ and 75% for L_2_ (Figure 6, Figure 7, Figure 8 and Figure 9). No or minor change was detected in unblocked or control areas.

At the contralateral thoracic control site, no significant differences in temperature were found between the different time points (*p* = 0.076). The recorded temperatures were 26.0 °C [24.3–33.3] (T_0_), 27.8 °C [25.0–33.0] (T_15_), 26.3 °C [24.9–35.3] (T_30_) and 26.4 °C [25.3–33.6] (T_45_), indicating a transient peak at T_15_.

## 4. Discussion

The present findings indicate that the rise in cutaneous temperature, attributable to vasodilation secondary to a successful nerve blockade, precedes a diminished or absent nociceptive response within the corresponding dermatome. These observations are consistent with evidence from human medicine [15], where similar temperature elevations have been reported across various locoregional techniques and anatomical sites. Notably, Zhang et al. [16] demonstrated that in humans, an increase in the delta T of the epidermis (ΔT_e_) higher than 0.63 °C within 15 min after thoracic paravertebral block was a reliable predictor of successful nerve blockade. In the present work, the temperature increase was significantly greater in blocked compared with unblocked dermatomes, and correlated with the absence of nociceptive response, supporting the hypothesis that thermography can serve as an indirect marker of effective blockade.

In contrast, some veterinary investigations have failed to reproduce these findings in dogs [17]. A plausible explanation is that Küls et al. [17] used bupivacaine, which has a latency of approximately 20 min, whereas outcome measurements were taken 2–11 min after injection, potentially preceding full onset. Moreover, those authors acknowledged as a limitation that block success was inferred from lack of intraoperative nociception under general anaesthesia while animals also received potent systemic analgesics, conditions under which a nociceptive response might be absent despite an incomplete block. The longer observation interval (15–45 min) and the use of lidocaine in our study, with a faster onset, may explain the more consistent association observed. Interestingly, although there was a tendency for blocked animals to exhibit higher temperatures during the haemostat pinch test, this difference was not statistically significant at 15 min but became significant at 30 and 45 min. This may be explained by the fact that some nerves required a longer time to achieve complete blockade, potentially due to a greater distance between the site of local anaesthetic deposition and the target nerve.

Thermographic assessment performed with a smartphone-compatible device appears sufficiently sensitive to detect temperature changes consistent with vasodilation. A technical limitation is the stated accuracy of ±3 °C, which could introduce measurement error. This risk may have been mitigated here by the sample size (12 animals) and by repeated measurements within animals. Nevertheless, clinicians should consider device accuracy when interpreting individual cases. Although other studies have employed higher-precision systems [24], the device used in the present work is attractive for routine practice due to its ease of use and substantially lower cost—features supported by its performance in human anaesthesia [25]. In short, FLIR^®^ One-type smartphone modules are great for screening and teaching, but they are markedly limited for quantitative research compared with ‘conventional’ research-grade thermal cameras. The main gaps are as follows: (1) Spatial resolution/IFOV: FLIR^®^ One sensors are 80 × 60 to 160 × 120 (4.8–19 k pixels), whereas research cameras commonly deliver 320 × 240, 640 × 480 or higher, yielding much finer temperature mapping and smaller spot sizes. (2) Thermal sensitivity (NETD) and accuracy: phone modules are typically around 70 mK and ±3 °C/±5%, while research units often achieve <40–50 mK and ±2 °C or ±2% with better calibration, enabling detection of subtler gradients. (3) Frame rate and radiometric video: FLIR^®^ One devices are limited to 8.7 Hz and often lack true radiometric video streaming, whereas research cameras commonly record calibrated radiometric video at 25–30 Hz for dynamic events. (4) Optics and controls: research cameras offer interchangeable lenses, precise manual focus, emissivity/reflected temperature control workflows, and stable onboard calibration; phone modules use fixed focus/short-focus optics and simplified apps, making rigorous emissivity/correction steps harder. (5) Stability and drift: low-cost modules can show greater sensor heating/drift over minutes, degrading quantitative repeatability versus pro systems [26,27]. Empirically, comparative studies in biomedical/veterinary settings report that while smartphone thermography can detect gross thermal changes, high-end cameras provide more reliable temperature estimates and better agreement with clinical endpoints. In a study in horses [21], both subjective and quantitative assessments of images captured with a FLIR^®^ P640 identified rises in cutaneous temperature over the median nerve dermatome following perineural anaesthesia. Although skin temperatures measured by the research-grade IR camera and a smartphone-based device were well-correlated, the smartphone unit tended to yield higher absolute values in horses, warranting caution. Moreover, the FLIR^®^ One registered temperature increases in both the treated and the contralateral limb, indicating it should not be relied upon to evaluate the success of a median nerve block. Future veterinary research should aim to define quantitative thresholds (ΔT cut-offs) and validate smartphone IRT against reference systems under controlled conditions.

Despite ultrasound guidance to maximise success, the observed proportions of decreased/absent nociceptive response were 71%, 91% and 75% for T_13_, L_1_ and L_2_, respectively. To the authors’ knowledge, the results obtained in our study can only be compared, with obvious limitations, to findings from cadaveric studies. Accordingly, our rates exceed those of the cadaveric study by Kramer et al. [13] using a similar ultrasound-guided approach. However, they are lower than those reported by Re et al. [14] for T_13_, higher for L_1_, and similar for L_2_; notably, Re et al. [14] employed a different sonographic technique. Nevertheless, it should be borne in mind that success rates reported in cadaveric studies are based on staining of the target nerve over at least a predefined length; however, true block success cannot be directly assessed, as the presence or absence of a nociceptive response cannot be evaluated. In contrast, the effectiveness of the block in our study is more clinically relevant, as it was performed in a live animal model, without the use of additional sedative drugs that could interfere with pain assessment, and in the presence of a nociceptive stimulus. Finally, an interesting observation was that, in one cow receiving L_1_–L_2_ blocks, the T_13_ dermatome showed no nociceptive response, and in another L_1_–L_2_ case, the L_3_ dermatome lacked a response. Potential explanations include individual anatomical variation, cranial/caudal spread of injectate, or inadvertent blockade of T_13_ or L_3_ when targeting L_1_ or L_2_. These findings might highlight inter-individual variation in nerve course and connective tissue planes and reinforce the importance of ultrasound guidance to ensure accurate deposition while recognising that spreading beyond the intended space is possible and sometimes advantageous.

A modest rise in temperature was seen across time points irrespective of the specific block performed. Although not statistically significant, several mechanisms could contribute: limited spread of effect to adjacent dermatomes; transient sympathetic alterations related to handling or stress; or environmental influences, since ambient temperature is known to affect bovine skin temperature [28]. The absence of significant variation in the contralateral thoracic control site, however, supports a genuine local physiological effect rather than a systemic or environmental artefact.

Lidocaine was selected for its favourable toxicity profile, rapid onset, moderate duration, and widespread use [8]. Withdrawal periods were irrelevant in this research setting but would be critical in production animals: lidocaine carries a 28-day meat and 7-day milk withdrawal. Additionally, the specific presentation used (Lidor) is not licenced for cattle and an authorised formulation would be required in clinical production settings.

Rapid and objective confirmation of successful regional anaesthesia remains a clinical challenge in large-animal practice, particularly when procedures must be performed promptly. IRT offers a non-contact, immediate feedback mechanism that could minimise unnecessary re-injections and enhance animal welfare. Furthermore, by providing objective evidence of effective blockades, IRT improves veterinary safety by reducing handling time and risk of exposure to a potentially stressed or reactive animal. However, clinicians must focus on relative temperature changes rather than absolute values and acknowledge the device’s inherent thermal accuracy limitations.

This study has limitations. First, although encompassing 12 cows and 24 nerve blocks, a larger sample would strengthen precision and generalisability. Second, the smartphone-based thermography system has not been extensively validated for this application in veterinary species. Third, no surgical procedure was undertaken, which would be the definitive test of complete block success. Fourth, thermographic values were taken directly from images without dedicated image-analysis software, potentially increasing measurement imprecision, again, partially offset by the within-subject repeated measures. Fifth, no control group was set up administering saline instead of lidocaine. Finally, ambient temperature was recorded only at baseline and not at 15, 30 and 45 min; unmeasured environmental fluctuations could have contributed to cutaneous temperature changes, particularly in non-blocked dermatomes that exhibited mild increases.

## 5. Conclusions

Smartphone-based infrared thermography (IRT) effectively detected cutaneous temperature increases associated with successful ultrasound-guided lumbar paravertebral blocks in cattle. Temperature rise correlated with loss of nociceptive response, supporting IRT as a rapid, non-invasive indicator of block success. Despite its lower accuracy compared to research-grade cameras, smartphone IRT showed sufficient sensitivity for clinical use under field conditions. Further validation under surgical scenarios and larger populations are needed to confirm its reliability in routine veterinary anaesthesia.

## Figures and Tables

**Table 1:** Skin temperature measurements over time according to the site at which the block was performed (T_13_, L_1_, L_2_, or L_3_). Temperature is presented as median [min–max]. ‘n’ indicates the number of cases in which the block was or was not performed. Note that no blocks were performed at L_3_, and the L_1_ nerve was always blocked. ‘NO’ indicates unblocked nerves and ‘YES’ indicates blocked nerves.

	No				Yes			
	T_0_	T_15_	T_30_	T_45_	T_0_	T_15_	T_30_	T_45_
Temp (°C) T_13_	n = 5	n = 5	n = 5	n = 5	n = 7	n = 7	n = 7	n = 7
	30.2 [26.3–31]	28.7 [28.6–35]	30.1 [27.1–33]	29.3 [27.8–32.2]	30 [28.4–33]	34 [32.4–36]	34.7 [32.5–35.3]	33.1 [26.5–35.5]
Temp (°C) L_1_	n = 0	n = 0	n = 0	n = 0	n = 12	n = 12	n = 12	n = 12
					30.6 [28.4–33]	34.2 [32.2–35.1]	34.6 [32.9–35.6]	33 [27–34.9]
Temp (°C) L_2_	n = 8	n = 8	n = 8	n = 8	n = 4	n = 4	n = 4	n = 4
	31.2 [28.8–32.6]	33 [31.6–34.9]	33 [32.1–34.1]	32.1 [31.1–33.3]	29.7 [28–30.6]	33.7 [32.4–34.6]	33.7 [32.6–34]	33.4 [32.6–34.1]
Temp (°C) L_3_	n = 12	n = 12	n = 12	n = 12	n = 0	n = 0	n = 0	n = 0
	30.2 [29–31.2]	32,7 [31.2–34.5]	32.2 [30.5–32.9]	31.9 [29.3–32.8]				

## Data Availability

Data supporting the reported results can be sent to anyone interested by contacting the corresponding author.

## References

[1] Grubb T., Lobprise H. (2020). Local and regional anaesthesia in dogs and cats: Overview of concepts and drugs (Part 1). Vet. Med. Sci..

[2] Margeti C., Kostakis C., Tsioli V., Karagianni K., Flouraki E. (2024). Local Anaesthesia Techniques in Dogs and Cats: A Review Study. Pets.

[3] Castejón-González A.C., Reiter A.M. (2019). Locoregional Anesthesia of the Head. Vet. Clin. North Am. Small Anim. Pract..

[4] Quesada N., d’Ovidio D., Read M., Monticelli P., Adami C. (2022). An Observational Survey Study on the Use of Locoregional Anaesthesia in Non-Conventional Species: Current Practice and Potential Future Developments. Animals.

[5] Campoy L. (2022). Development of Enhanced Recovery After Surgery (ERAS) protocols in veterinary medicine through a one-health approach: The role of anesthesia and locoregional techniques. J. Am. Vet. Med. Assoc..

[6] Edmondson M.A. (2008). Local and regional anesthesia in cattle. Vet. Clin. North Am. Food Anim. Pract..

[7] Weaver A.D., Atkinson O., St. Jean G., Steiner A. (2018). General considerations and anaesthesia. Bovine surgery and lameness.

[8] Valverde A., Lamont L.A., Grimm K.A., Robertson S.A., Love L., Schroeder C. (2024). Ruminant and swine local anesthetic and analgesic techniques. Veterinary Anesthesia and Analgesia.

[9] Kelton D.F., Lissemore K.D., Martin R.E. (1998). Recommendations for recording and calculating the incidence of selected clinical diseases of dairy cattle. J. Dairy Sci..

[10] USDA Dairy Cattle Management Practices in the United States, 2014. https://www.aphis.usda.gov/sites/default/files/dairy14_dr_parti_1.pdf.

[11] Sexton M., Buckley W., Ryan E. (2007). A study of 54 cases of left displacement of the abomasum: February to July 2005. Ir. Vet J..

[12] Rioja E., Lamont L.A., Grimm K.A., Robertson S.A., Love L., Schroeder C. (2024). Local anesthetics. Veterinary Anesthesia and Analgesia.

[13] Kramer A.H., Doherr M.G., Stoffel M.H., Steiner A., Spadavecchia C. (2014). Ultrasound-guided proximal paravertebral anaesthesia in cattle. Vet. Anaesth. Analg..

[14] Re M., Blanco-Murcia J., Villaescusa A., De Gaspar I., de Segura I.A.G. (2016). Comparison of paravertebral blockade techniques with and without ultrasound guidance in calves. Am. J. Vet. Res..

[15] Lange K.H., Jansen T., Asghar S., Kristensen P.L., Skjønnemand M., Nørgaard P. (2011). Skin temperature measured by infrared thermography after specific ultrasound-guided blocking of the musculocutaneous, radial, ulnar, and median nerves in the upper extremity. Br. J. Anaesth..

[16] Zhang S., Liu Y., Liu X., Liu T., Li P., Mei W. (2021). Infrared thermography for assessment of thoracic paravertebral block: A prospective observational study. BMC Anesthesiol..

[17] Küls N., Blissitt K.J., Shaw D.J., Schöffmann G., Clutton R.E. (2017). Thermography as an early predictive measurement for evaluating epidural and femoral-sciatic block success in dogs. Vet. Anaesth. Analg..

[18] Gamal M., Hasanin A., Adly N., Mostafa M., Yonis A.M., Rady A., Abdallah N.M., Ibrahim M., Elsayad M. (2023). Thermal Imaging to Predict Failed Supraclavicular Brachial Plexus Block: A Prospective Observational Study. Local Reg. Anesth..

[19] Eddy A.L., Van Hoogmoed L.M., Snyder J.R. (2001). The role of thermography in the management of equine lameness. Vet. J..

[20] Casas-Alvarado A., Mota-Rojas D., Hernández-Ávalos I., Mora-Medina P., Olmos-Hernández A., Verduzco-Mendoza A., Reyes-Sotelo B., Martínez-Burnes J. (2020). Advances in infrared thermography: Surgical aspects, vascular changes, and pain monitoring in veterinary medicine. J. Therm. Biol..

[21] Bernard V., Staffa E., Pokorná J., Šimo A. (2025). Assessing detector stability and image quality of thermal cameras on smartphones for medical applications: A comparative study. Med. Biol. Eng. Comput..

[22] Agne G.F., Adamson K., McGlinchey L., Kravchuk O., Santos L., Schumacher J. (2024). Comparison of a hand-held high-end resolution infrared thermography (FLIR P640) and a smartphone infrared thermographic device (FLIR One) for the assessment of skin surface temperature after anaesthetising the median nerve in Healthy horses. PLoS ONE.

[23] du Sert N.P., Hurst V., Ahluwalia A., Alam S., Avey M.T., Baker M., Browne W.J., Clark A., Cuthill I.C., Dirnagl U. (2020). The ARRIVE guidelines 2.0: Updated guidelines for reporting animal research. PLoS Biol..

[24] Małyski M., Rustecki B., Kalicki B., Jung A. (2015). Thermal imaging evaluation of paravertebral block for mastectomy in a high-risk patient: Case report. J. Clin. Monit. Comput..

[25] Bruins A.A., Kistemaker K.R.J., Boom A., Klaessens J.H.G.M., Verdaasdonk R.M., Boer C. (2018). Thermographic skin temperature measurement compared with cold sensation in predicting the efficacy and distribution of epidural anesthesia. J. Clin. Monit. Comput..

[26] Obinah M.P.B., Nielsen M., Hölmich L.R. (2020). High-end versus Low-end Thermal Imaging for Detection of Arterial Perforators. Plast. Reconstr. Surg. Glob. Open.

[27] Kirimtat A., Krejcar O., Selamat A., Herrera-Viedma E. (2020). FLIR vs SEEK thermal cameras in biomedicine: Comparative diagnosis through infrared thermography. BMC Bioinform..

[28] Lysyk T.J. (2008). Effects of ambient temperature and cattle skin temperature on engorgement of *Dermacentor andersoni*. J. Med. Entomol..

